# Wide Ranging Insect Infestation of the Pioneer Mangrove *Sonneratia alba* by Two Insect Species along the Kenyan Coast

**DOI:** 10.1371/journal.pone.0154849

**Published:** 2016-05-12

**Authors:** Elisha Mrabu Jenoh, Elisabeth M. R. Robert, Ingo Lehmann, Esther Kioko, Jared O. Bosire, Noah Ngisiange, Farid Dahdouh-Guebas, Nico Koedam

**Affiliations:** 1 Kenya Marine and Fisheries Research Institute (KMFRI), P.O. Box 81651, Mombasa, Kenya; 2 Laboratory of Plant Biology and Nature Management (APNA), Vrije Universiteit Brussel, Pleinlaan 2, B-1050, Brussels, Belgium; 3 Laboratory of Systems Ecology and Resource Management, Département de Biologie des Organismes, Université Libre de Bruxelles (ULB), CP 169, Avenue Franklin D. Roosevelt 50, CPI 264/1, B-1050, Brussels, Belgium; 4 Laboratory for Wood Biology and Xylarium, Royal Museum for Central Africa (RMCA), Leuvensesteenweg 13, 3080, Tervuren, Belgium; 5 University of Bonn, Zoological Research Museum, Alexander Koenig (ZFMK) Adenauerallee 160, 53113, Bonn, Germany; 6 National Museums of Kenya (NMK), P.O. Box 40658–00100, Nairobi, Kenya; Institute of Plant Physiology and Ecology, CHINA

## Abstract

Insect infestation of mangroves currently threatens mangrove forest health and management. In the Western Indian Ocean region, little is known about insect damage to mangroves despite the fact that numerous infestations have occurred. In Kenya, infestations of *Sonneratia alba* have persisted for almost two decades, yet the taxonomic identity of the infesting pest(s), the extent of infestation, the pests’ biology, the impacts of infestation on host and the ecosystem, the host’s defensive strategies to the infestation are poorly understood. *S*. *alba* is a ubiquitous, pioneer mangrove species of the Indo-Pacific, occurring along the waterfront in a variety of mangrove ecosystem settings. Our main objectives were to identify the pest(s) responsible for the current dieback of *S*. *alba* in Kenya, and to determine the extent of infestation. To identify the pests responsible for infestation, we trapped emergent insects and reared larvae in the laboratory. To determine the overall extent of infestation within the *S*. *alba* zone, we assessed nine sites along the entire Kenyan coastline for the presence or absence of infested mangroves. Insect infestation in two mangrove embayments (Gazi and Mida) was quantified in depth. Two wood-boring insects were identified: a metarbelid moth (Lepidoptera, Cossoidea) of undescribed genus and the beetle *Bottegia rubra* (Cerambycidae, Lamiinae).The metarbelid moth infests mangroves in both northern (from Ngomeni to Kiunga) and southern regions (from Vanga to Mtwapa) of the Kenyan coast. *B*. *rubra* appeared in low density in Gazi, and in high density in Mida, Kilifi, and Ngomeni, with densities gradually decreasing northward. Insect infestation levels reached 18% in Gazi and 25% of *S*. *alba* stands in Mida. Our results indicate that *B*. *rubra* has the ability to infest young mangrove trees and expand its range, posing a danger to rehabilitation efforts where plantations have been established. Thus, there is great need for forest managers to address the recent increased levels of infestation in Kenyan mangroves; apart from the ecological interest such plant-herbivore relations bring in this ecosystem.

## Introduction

Biotic interactions such as insect infestations may damage organisms but they also play their role in ecosystem processes [1,2,3,4,5]. Insect defoliators and woodborers, including beetles, moths, and horntail wasps, are widely recognized as agents of ecosystem disturbance that can influence forest production and nutrient cycling. Insect infestations can create large-scale forest diebacks [6,7,8,9,10]. Infesting insects feed and make their homes on bark, trunks, and branches of trees and shrubs. Insect borers damage plants by tunneling through the inner bark and cambium; if the stem is completely girdled, the plant dies at or above the damaged site. If the tree is weakened structurally, leaves and branches may fall [11]. Insect damage can severely affect the quality of timber, and render trees susceptible to disease and secondary fungal infestation. As resources are reallocated to compensate for herbivore damage, the productive capacity of a tree may be reduced [12,13]. Insect infestation is a scientifically neglected problem in mangrove research and management [14,15,16]. This is partly due to the perception that the tannins in mangroves render them inedible to most herbivorous insects [17], and that tidal flooding inhibits insect population growth. Consequently, insects that attack mangroves are understudied, with a few exceptions [18,19,20,21,22,23,24,25,26]. Insect infestations in mangrove forests in the Western Indian Ocean (WIO) region have not been well-documented, though several outbreaks have occurred in this area. For instance, in 2003, substantial stands of *Rhizophora mucronata* Lamk. were defoliated in the Pemba region of Tanzania. In Kiunga and Lamu, where the largest contiguous mangrove forests of Kenya are found, an unknown insect pest is thought to be responsible for the dieback of *R*. *mucronata* over the last two decades. However, this infestation has never been studied. A 2005 survey reported a decline of *Sonneratia alba* J. Smith due to an insect infestation that rapidly spread northwards towards the mangroves of Somalia [14]. This infestation was attributed to a cerambycid beetle and a metarbelid moth, the taxonomic status of which is currently under revision [27,28]. In Kenya, these insects are reported to have an overlapping geographical range, but they appear in different locations on the host trees [29].

The symptoms of infestation in *S*. *alba* populations suggested that more than one insect species infests *S*. *alba* [14,29]. To date, there exist insufficient and conflicting reports on the identity of these insects [14,30,31], their spatial distribution and biology, and the extent and severity of infestation. This study reports on the identity of the insects, their distribution along the Kenyan coast, and the level and extent of the infestation of *S*. *alba* populations. It is hoped that this information will contribute to the development of management and conservation strategies for the East African mangrove ecosystem and generate further insight into plant-herbivore relations in these particular assemblages.

## Materials and Methods

### Ethics Statement

The Kenya Marine and Fisheries Research Institute (KMFRI) is a state corporation in the Ministry of Agriculture Livestock and Fisheries, with a mandate to conduct aquatic research covering all Kenyan waters and corresponding riparian areas, including Kenya’s exclusive economic zone (EEZ) in the Indian Ocean waters. The corresponding author is a KMFRI employee and had full permission to carry out this study.

The authors declare that no endangered species or vertebrates were involved in this research.

### Study Sites

**Fig 1:**
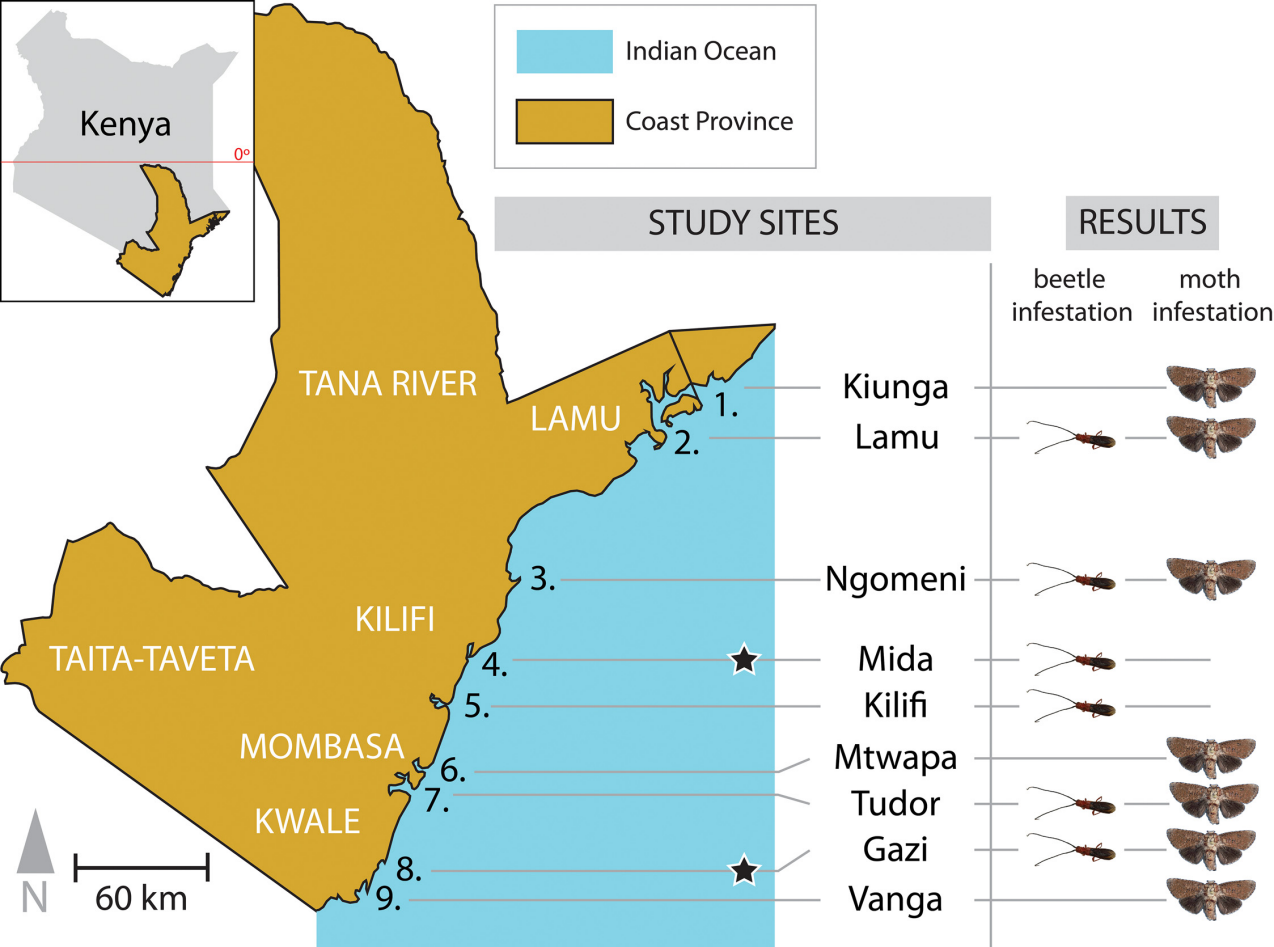
Map of Kenya highlighting the Kenyan coast region and the distribution of the infesting insects (the metarbelid moth and *Bottegia rubra*) in the surveyed sites.

Mangrove forests in Kenya cover approximately 54,000 ha, most of which are in the Lamu and Tana River counties [32, 33] (Fig 1). According to Dahdouh-Guebas (2000), Kairo (2001), Fao (2007), Spalding (2010) and Kirui (2012), [34,35,36,37,38] there are nine mangrove species in Kenya; the dominant species are usually *Rhizophora mucronata* and *Ceriops tagal* (Perr) C.B. Robinson. Mangrove forests in Kenya frequently display the typical zonation pattern of mangroves in eastern Africa: the seaward side is occupied by the *Sonneratia-Rhizophora*-*Avicennia* (tall) assemblage, followed by *Rhizophora-Bruguiera-Ceriops* in the middle zone and the *Avicennia-Lumnitzera-Xylocarpus* complex with often dwarf *Avicennia* on the landward side [35,39,40].

This study involved (i) a detailed study consisting of (a) trapping and rearing larval and pupal stages of woodborers to an adult stage for taxonomic classification, (b) scoring of the *S*. *alba* forest to determine the level of infestation, and (c) determining the lowest level of infestation in Gazi and Mida so as to confirm differences in the spatial distribution between the infesting insects; and (ii) a survey done along the Kenyan coast (Fig 1) to determine the extent of the infestation within Kenya.

#### Detailed study site 1: Gazi

The first detailed study was conducted in Gazi (Kenya) approximately 55 km south of Mombasa, in Kwale County (04°44′S 039°51′E) (Fig 1). All nine mangrove species [41] in Kenya are present at Gazi, *R*. *mucronata*, *Ceriops tagal*, and *Avicennia marina* being dominant in various assemblages. In addition to anthropogenic degradation such as overharvesting of mangroves for fuel wood, timber and fish traps, clearing and conversion to other land uses such as agriculture, aquaculture, urban development, tourism and salt production and pollution of mangroves in Kenya [42,43,44], *S*. *alba* is experiencing a dieback due to sedimentation and silting [30,45,46]. For almost two decades, insect damage has also led to the loss of infested *S*. *alba* branches and trees [14,46]. In October 1991, a project was initiated to restore the mangroves in Gazi by replanting various tree species, including *S*. *alba* seedlings [47]. Thus, allowing a comparison of infestation levels in natural and replanted mangrove populations. Four sampling plots were established in similar, relatively dense *S*. *alba* forest: Plot A (04°25′901′′S, 039°30′676′′E), Plot B (04°25′589′′S, 039°30′789′′E), Plot C (03°21′100′′S, 039°58′124′′E), and Plot D (03°21′100′′S, 039°58′124′′E). In these sites and Kenya in general, *S*. *alba* comprises a small proportion of the mangrove stands [35,48], however, this species is frequently dominant at the seaward edge and on mudflats. Plot A consisted of two sections, one with large, old *S*. *alba* trees, and the other consisted of eight-year-old and five-year-old replanted *S*. *alba* trees. Plot B was composed mainly of old *S*. *alba* trees. This plot has been affected by sedimentation and silting up and hence lost several trees. Plot C was composed of fifteen-year-old replanted *S*. *alba* trees. Plot D was composed of a mature, naturally grown *S*. *alba* stand located inside the main water channel in Gazi.

#### Detailed study site 2: Mida

The second detailed study was conducted in Mida, located 25 km south of Malindi and 100 km north of Mombasa (03°34′S, 039°96′E) (Fig 1). The study site was chosen for its high density of beetle infestation, and because initial studies on this infestation were conducted there [14,29]. Mida Creek lies in a planigraphic area of 31.6 km^2^ [49] that is under government protection for conservation purposes [50]. In order to investigate the extent of infestation in the mangroves of Mida, plots were laid following the same design as in the first detailed study site in Gazi. However, since the *S*. *alba* forest of Mida forms a narrow band, sampling was restricted to two plots: Plot A, containing narrow band of older trees, and Plot B, containing relatively young *S*. *alba*.

### Specimen Collection and Identification

**Fig 2:**
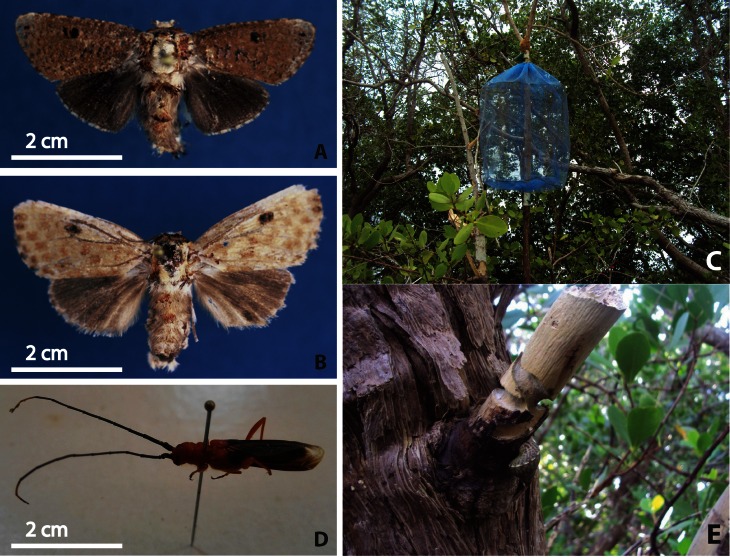
The two woodborer species infesting *S*. *alba* in Kenya.

To associate the larvae borers to the adults and to use the adult insects for taxonomic identification purposes, improvised traps and rearing of larvae and pupae were employed for the metarbelid moth in Gazi and rearing for the cerambycid beetle in Mida. Trees infested by moth larvae were identified by the presence of reddish-brown frass in areas on the bark near the infestation. Fifteen traps were then placed to collect adult insects emerging from the branches (Fig 2C). The traps provided enough space for the wings of adults to expand and dry normally [51]. Other branches with active signs of insect activity and dead branches were dissected in order to collect larvae. Larvae were kept in perforated plastic containers. Fresh branches were provided biweekly for food and shelter. These containers were maintained in darkness at the Kenya Marine and Fisheries Research Institute laboratory in Mombasa, located in a coastal site (Indian Ocean) with similar temperature and humidity to the insects’ native habitat. Pupation and emergence dates were recorded, and adult males were placed in containers with adult females until the females laid eggs.

To trap the beetles, recently infested branches (identified by presence of wilting, drying and discoloring of leaves and a deep red band around the infested branch) were cut 150 cm below the start of the dry part and taken to the Kenya Marine and Fisheries Research Institute laboratory in Mombasa for rearing. Adults were collected upon emergence. Adult insects were killed by placing them in a jar containing ethyl acetate [52]. Pinned specimens were transported to the invertebrates section of the National Museums of Kenya in Nairobi for systematic identification and storage. The specimens are deposited at this institution.

#### Taxonomic identification

Identification of the moths was performed by one of us (IL), by comparing the specimens from Kenya with *ca*. 550 species from major natural history museums in Europe and Africa as well as private collections representing the Afrotropical Region as well as India, Nepal, and Southeast Asia. Identification of the cerambycid beetle was conducted with keys by Booth (1972) [53] and the reference insect collection at the National Museums of Kenya.

### Quantification and vertical distribution of damage

Ten-meter transects parallel to the shoreline in the *S*. *alba* zone in Gazi and in Mida were used as sampling sub-plots. Furthermore, 10 m gaps were maintained between sub-plots to ensure independence of samples. Sampling plots were composed of 9–12 sub-plots in Gazi and 7 and 9 sub-plots in Mida. To quantify insect damage, two observers moved perpendicularly 10 m far from the sampling plot to record the general health of the sampling sub-plot using a modified version of the method of Feller and McKee (1999) [24]. Observers answered the following questions: (i) Is the canopy completely healthy?; (ii) Is there any sign of insect damage?; (iii) Is there any dry branch present and clearly seen on the canopy?; (iv) What is the percentage damage as seen from the sub-plot?; (v) Are there any plant species other than *S*. *alba* seen in the canopy?; (vi) Are there other infested mangrove tree species in the sub-plot?; (vii) Are there *S*. *Alba* seedlings in the sub-plot? Finally (viii) Which species of seedlings are present in the sub-plot? Other independent observers recorded the number of trees in the plot, the number of species, the number of old infestation sites in *S*. *alba* trees (diagnosed by large amounts of large, dark brown frass granular material), and the number of new infestation sites (diagnosed by small amounts of small, light-brown frass grains).

Some studies have suggested that tree size could be a factor affecting insect’s spatial distribution and colonization range [29]. The shortest distance from the ground to the first infested branch in 62 randomly picked trees in Mida and Gazi was therefore measured in order to understand the vertical distribution of these two woodborers.

### Coastline Survey

A survey of the *S*. *alba* mangrove forest zone was done from the Southern to the Northern mangroves of Kenya in Kiunga (01°74′S 041°49′E), Lamu (02°17′S 040°55′E), Ngomeni (02°99′S 040°17′E), Mida (03°34′S039°96′E), Kilifi (03°63′S039°75′E), Mtwapa (03°95′S 039°75′E), Tudor (04°03′S 039°66′E), Gazi (04°43′S 039°50′E), and Vanga (04°66′S 039°12′E), in order to determine the extent of the infestation of *S*. *alba* along the Kenyan coastline (Fig 1). For each location, researchers walked in and along the *S*. *alba* forest zone and recorded the presence or absence of woodborers, based on the species-specific products (frass, exuviae) and effects (galleries, exit holes) of infestation [14].

### Statistical Analyses

Data on percentage health of the sub-plots, percentage infestation level of the sub- plots, and lowest infestation height of the infested trees in Gazi and Mida were analyzed using STATISTICA 8 (Statsoft, TULSA, USA). Data were checked for normality before using a one way Analysis of Variance (ANOVA) to confirm trends between sampling plots. Levene’s test was used to ensure that the data met the assumptions for ANOVA. Significance was assessed at α = 0.05.

## Results

### Rearing and Identification of Adults

From the fifteen traps (Fig 2C) placed in the *S*. *alba* forest in Gazi, thirteen adult moths were collected. However, the trapped adults lost considerable proportions of the scales on their wings as they attempted to escape from the trap, rendering them unsuitable for identification purposes. Therefore, adults from lab-reared larvae and pupae were used for taxonomic identification.

**Fig 3:**
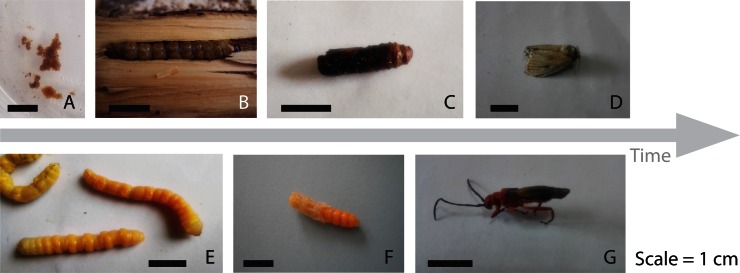
Growth stages of the two insect pests infesting on *S*. *alba*.

Larvae obtained from the field appeared as two types: the first was large with a creamy brown colour and the second was smaller and darker. These larvae produced two types of pupa the first type eventually produced larger, whitish-grey adults, and the second type produced darker, smaller adults. The larger adults produced 200–650 eggs (n = 67) within 12 h of eclosion Fig 3A. The small dark adults did not produce eggs. In the laboratory, the pupal period was 28–32 days (n = 95). The adult lifespan was 7–8 days (n = 95).

The moth is an undescribed species close to *Salagena albicilia* (Hampson), a metarbelid moth that occurs in moist woodlands and forests of southern Malawi (Fig 2A and 2B). The ongoing revision of the family Metarbelidae shows that there are at least five other species that belong to the same genus occurring in southeastern Ghana (west Africa), western Kenya, southwestern and eastern Tanzania, northern Namibia, and northeastern South Africa, in various lowland and montane woodland or forest habitats (Lehmann, in prep.). All five species and the genus are currently undescribed.

Beetle larvae from Mida took 29–36 days to reach the pupal stage, which lasted 6–8 days (n = 56) (Fig 3B). Male and female adults were not distinctly different. Some adults laid eggs 3–4 days after emerging. The beetle was identified as *Bottegia rubra* (Cerambycidae: Lamiinae) [54] (Fig 2D).

### Infestation Levels

**Fig 4:**
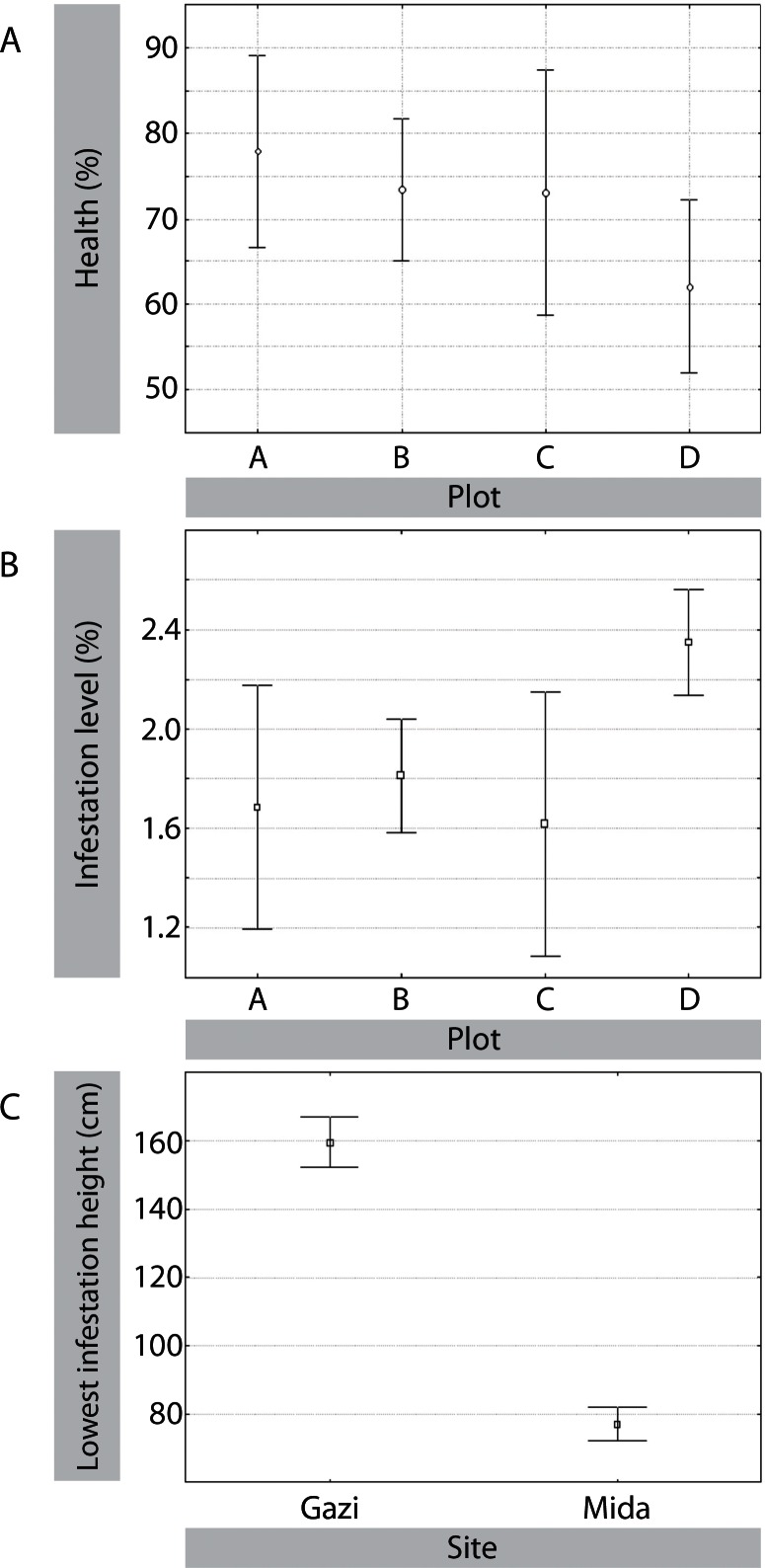
Degree and lowest height of infestation on *S*. *alba* by the metarbelid moth and *B*. *rubra* in Gazi and Mida.

All sub-plots scored showed signs of insect infestation. There was no sub-plot that was either entirely dead or entirely healthy in both Gazi and Mida (Fig 4A). Overall, 18% of the *S*. *alba* forest in Gazi was infested. All plots in Gazi were also infested by a parasitic mistletoe-type plant; *Agelanthus* cf. *kayseri* (Engler.). Observations from inside the sub-plots revealed that all trees in all plots had been infested. Old infestations were more numerous than new infestations in all plots (Table 1). Infestation level in Gazi varied significantly among plots (*F*_3,34_ = 5.183; *P*< 0.001); it was highest in plot D, followed by B, and then by A and C (Fig 4B). In Mida, plot A had a higher average health than plot B (90.0% vs. 69.3%; t_13_ = 2.6;*P*<0.01) and a lower average infestation level (10.4% vs. 39.6%; t_13_ = 2.6; *P*< 0.01),

**Table 1:** Infestation of the four sampling sites in Gazi. The figures are percentage totals ± standard deviation of new and old infestation inside the sampling plots. No species other than *S*. *alba* were infested in any of the studied plots.

Plots	New infestation	Old infestation
A	38.64 ±4.2	61.36 ±9.7
B	36.93 ±2.9	63.08 ±5.5
C	**21.92 ±3.4**	**78.08 ±15.5**
D	37.91 ±3.8	62.09 ±8.09

### Lowest infestation height

Average lowest infestation height above ground level was higher in Gazi than in Mida (159.6cm, n = 42 vs. 77.1cm, n = 62; (F_2, 77_ = 153.105; *P*<0.001). The lowest infestation heights above ground level recorded for Gazi and Mida were 122.5 and 41 cm, respectively (Fig 4C).

### Distribution of infestation along the Kenyan coastline

The two woodborers had different spatial niche distributions along the Kenyan coast (Fig 1). In areas where their ranges overlapped, such as Gazi, Tudor, Ngomeni, and Lamu, *B*. *rubra* was found in very low density as compared to the undescribed metarbelid moth. *B*. *rubra* colonized mainly Mida and Kilifi, as the sole woodborer infesting *S*. *alba*. In Gazi, Tudor, Ngomeni, and Lamu, *B*. *rubra* appeared in very few trees and in few branches within those trees. In Vanga, Gazi, Tudor, Mtwapa, Ngomeni, and Lamu, *S*. *alba* was mainly infested by the undescribed metarbelid moth (which was not found from Kilifi to Mida) (Fig 1). Interestingly, during extensive field studies in the Shimoni forest from 2005 to 2008, the metarbelid moth could not be found in this drier coral rag forest occurring close to the shoreline, just a few hundred meters from mangrove stands (Ingo Lehmann, pers. obs.).

## Discussion

### Insect Identification

Laboratory rearing of larvae proved to be the most reliable way of obtaining samples suitable for taxonomic identification. This species was previously identified as *Salagena discata* Gaede [14] and as *Salagena obsolescens* (Hampson) [29,30] but proved to be a species of a hitherto undescribed genus with closely related species in western, eastern, and southern Africa.

### Survey of *S*. *alba* Forest Stand along the Kenyan Coastline

Although the metarbelid moth and *B*. *rubra* attack the same host, there is a north-to-south distribution gradient, with the two species dominating in different sites. The factors contributing to this gradient are not yet fully understood and no data are as yet available for neighboring coasts of Somalia or Tanzania. However, it seems that *B*. *rubra* has an affinity for relatively young and short *S*. *alba* trees like those found in Mida, whereas the metarbelid moth does not (Fig 4C). Even where the two woodborers co-exist, *B*. *rubra* attacks the shorter and younger trees. In Gazi, for example, they are found mainly on the *S*. *alba* fifteen years old plantation, whereas they are almost absent in the old *S*. *alba* forest. In the few occurrences where *B*. *rubra* attacked large, mature trees, they attacked the lower branches within the tree. Because the moth is found mostly in the upper branches, this leads to a spatial separation between the two pest species, even when they are attacking the same individual tree (Fig 4C). It is possible that this divergence is an adaptation reducing interspecific competition. Because the two species attack different parts of the tree and therefore experience little competition even when on the same host, the geographic gradient in their abundance is probably not due to interspecific competition, and is more likely explained by other environmental or dispersal mechanisms, or other factors, e.g. related to a host’s response.

**Fig 5:**
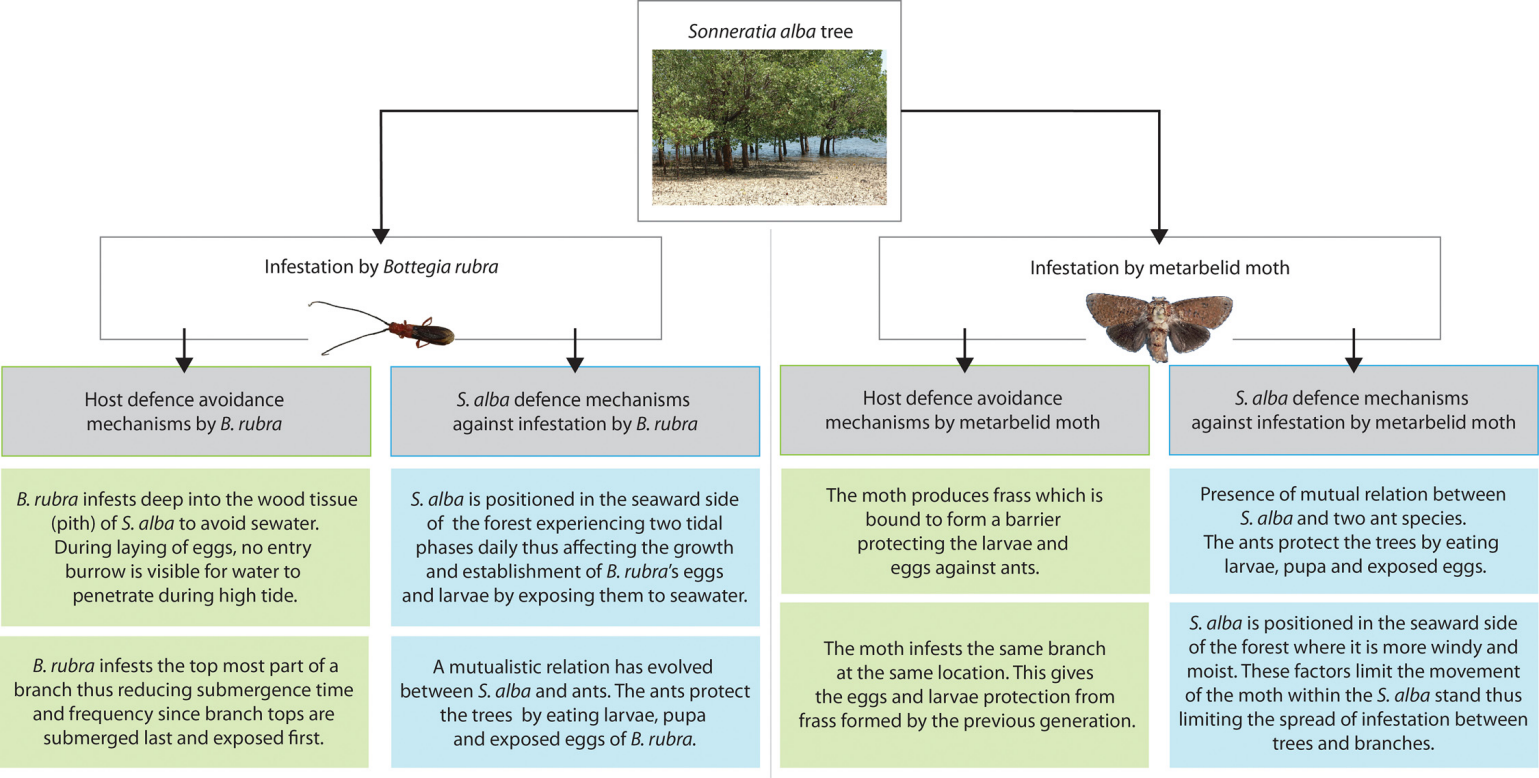
Scheme diagram of the hosts’ protection mechanisms against infestation by the insect pests.

The preference of *B*. *rubra* at the lower parts of *S*. *alba* is interesting for multiple reasons. Young, short trees experience longer submergence than taller, mature trees. Long hours of submergence increase the likelihood that larvae and pupae may drown or asphyxiate, making tidal amplitude and height above datum important factors in the survival of *B*. *rubra*. However, because *B*. *rubra* leaves no visible entry hole on infested branches, water does not penetrate these branches while they are submerged. Additionally, *B*. *rubra* larvae live and eat in the deep tissues of infested branches, where they are shielded from the tide. Fig 5

**Fig 6:**
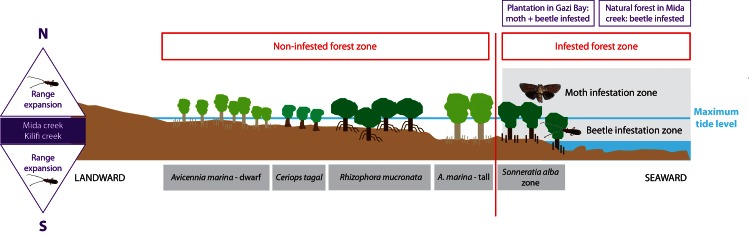
Scheme indicating the major and common mangrove zones, with the tidal range and presence of insect infestation of both metarbelid moth and *B*. *rubra*, as observed along the Kenyan coast.

Infestation of lower branches allows *B*. *rubra* to avoid predators like ants, which forage on larvae and pupae of wood boring insects. Submergence naturally protects the beetles from such predators Fig 6. The metarbelid moth has evolved different defensive strategies; including girdling a network of galleries in a host branch and the release of offensive compounds which slow and deter attacking ants as the larva changes its location to another gallery as well as sealing it from the ant-infested gallery. Fig 5

Our findings are similar to those of Gordon and Maes (2003) [29], who attributed the distribution patterns of these pests to the fact that in Mida, *S*. *alba* mangroves are smaller than in Gazi and most forests in the south coast. Further studies to understand the causes of this distribution gradient are needed. It is also not known why the metarbelid moth does not occur in Kilifi and Mida despite the existence of large *S*. *alba* trees in fairly extensive assemblages in those areas.

### Extent of Infestation Damage

In Gazi, no scored plot was free from infestation. Even though we recorded an average of 18% visible infestation in Gazi, close examination revealed that all trees had been infested, even though not all branches showed signs of damage, i.e. defoliation and dried leaves. This suggests a much more weakened *S*. *alba* forest than is visible on general observation of the sub-plots. It seems that the parasitic plant *Agelanthus* cf. *kayseri* also contributes to the weakening of *S*. *alba* by drawing of nutrients, water, possibly carbohydrates, thus the damage to the forest is a combination of infestation by multiple species. All plots had *S*. *alba*, *A*. *marina* and *R*. *mucronata* seedlings present suggesting that insect infestation does not directly interfere with seed dispersal and recruitment of seedlings even though defoliation and drying of branches could be reducing the amount of fruits, propagules and flowers produced by a single branch therefore impacting on the overall recruitment of seedlings.

Plot D in Gazi, which registered the highest infestation level, had both young and old naturally-grown trees. Such a forest is a structurally complex biotope (in terms of different height and ages of the *S*. *alba* trees), which favors successful colonization and multiplication of both insects. Both plots A and B had young plantations of *S*. *alba* trees which were either not fully infested or were just beginning to be infested by *B*. *rubra* also. Despite the plantations in plots A, B and C having reached an appropriate size for infestation by *B*. *rubra* much earlier, they are just beginning to be infested. This suggests that *B*. *rubra* only recently expanded its range to the south. As infestation of *S*. *alba* has generally increased in the recent past, *B*. *rubra* seems to be expanding its range, remaining specific to this host species in the whole region. Because it tends to attack small trees, including young trees in plantations, this insect poses a challenge to mangrove restoration efforts. Therefore, it is important that it be given priority in forest management.

Plot C was the least infested of the plots. This could be because infestation by *B*. *rubra* was only beginning. Because this plot had the highest number of old moth infestations, but the lowest number of new moth infestations (Table 1), it appears that most old infestations in this plantation did not lead to the establishment of new ones. This could be due to a lack of branches of the appropriate size for establishment by young larvae; the branches of trees in this plantation are thin compared to those of natural forest trees. It is also possible that conditions where eggs are laid are different in the plantation than in a natural mangrove forest. Evenly spaced plantation has few and thin side branches which leads to sparse foliage in the plantation. Such a forest could be exposing the frass where the eggs are laid to more elevated moisture than is the case in a natural forest where the numerous leaves reduce the moisture reaching the frass thus affecting the viability of the eggs. Based on our field observations, emerging adult moths lay their eggs in the old frass from which they emerged. However, this mechanism by which the population of the metarbelid moth may be reduced in plantations requires further investigation.

The fact that the moth experiences different success in this plantation compared to the natural forest in part does not align with the finding by Bosire (2004) [55] that rehabilitated mangroves support faunal recolonization and thus are similar to natural sites in supporting biodiversity. Our finding suggests that forest structure, not simply the presence of mangroves, is important for the full recovery of a mangrove forest ecosystem. It is important to note that unlike our work, Bosire’s (2004) work was conducted mainly on marine benthic organisms.

Further, since the plantation does not manifest full infestation characteristics such as defoliation, it suggests that *B*. *rubra* had not extended its range to Gazi by the time this plantation was old enough to be infested, or the densities of *B*. *rubra* were still too low to render a visible manifestation of infestation. This conclusion is supported by the fact that even though plots A and B included plantation trees that were of reasonable height for infestation by *B*. *rubra*, the infested trees were still few and the infestation just beginning to occur. This suggests that *B*. *rubra* has only recently extended its range in Gazi and along the south coast of Kenya.

In Mida, differences in the height of the *S*. *alba* trees appear to have led to the significant difference in the level of infestation. This further emphasizes the height preference of the beetle for the trees it infests.

## Conclusion

In this study, we identified two woodborers infesting *S*. *alba* forests in Kenya as *B*. *rubra* (cerambycid beetle) and an undescribed metarbelid moth, the genus of which is also undescribed. We report the first success in rearing the metarbelid moth from larvae to adult, providing the opportunity for successful description and taxonomic identification and use of morphological traits to determine the distinction between the males and females from the pupal stage. The occurrence of *B*. *rubra* in parts of the south and north coast of Kenyan suggests range expansion (Fig 6). We suggest priority be given to studying *B*. *rubra* since it has a high capacity for dispersal and the ability to infest young mangroves, thus posing a danger to rehabilitation efforts where plantations have been established. Factors leading to the spatial geographic distribution of the beetle and that of the metarbelid moth in *S*. *alba* forests require further investigation. In addition to establishing the moth’s full life cycle, research into the impacts of both insects on the ecophysiology and the wood anatomy of host tree, as well as on their ecological role in the mangrove ecosystem, is sorely needed, including what predators they may have and how they contribute to the food web. Future studies should also consider whether *S*. *alba* plantations reduce the population of the metarbelid moth, or act as a reservoir for it, and thus apply pressure to nearby natural stands. The occurrence of a host-specific woodboring moth of a wide-ranging mangrove species host and the fact that it belongs to a newly discovered genus merits evolutionary and biogeographic consideration.
